# Household preferences and willingness to pay for health insurance in Kampala City: a discrete choice experiment

**DOI:** 10.1186/s12962-021-00274-8

**Published:** 2021-04-20

**Authors:** Edward Kalyango, Rornald Muhumuza Kananura, Elizabeth Ekirapa Kiracho

**Affiliations:** 1 Department of Health Policy and Planning, School of Public Health, College of Health Sciences, Makerere University, Kampala, Uganda; 2 Department of international Development, The London School of Economics and Political Science, London, UK

**Keywords:** Health insurance, Discrete choice experiment, Preferences, Willingness to pay, Kampala, Uganda

## Abstract

**Introduction:**

Uganda is in discussions to introduce a national health insurance scheme. However, there is a paucity of information on household preferences and willingness to pay for health insurance attributes that may guide the design of an acceptable health insurance scheme. Our study sought to assess household preferences and willingness to pay for health insurance in Kampala city using a discrete choice experiment.

**Methods:**

This study was conducted from 16th February 2020 to 10th April 2020 on 240 households in the Kawempe division of Kampala city stratified into slum and non-slum communities in order to get a representative sample of the area. We purposively selected the communities that represented slum and non-slum communities and thereafter applied systematic sampling in the selection of the households that participated in the study from each of the communities. Four household and policy-relevant attributes were used in the experimental design of the study. Each respondent attended to 9 binary choice sets of health insurance plans that included one fixed choice set. Data were analyzed using mixed logit models.

**Results:**

Households in both the non-slum and slum communities had a high preference for health insurance plans that included both private and public health care providers as compared to plans that included public health care providers only (non-slum coefficient β = 0.81, P < 0.05; slum β = 0.87, p < 0.05) and; health insurance plans that covered extended family members as compared to plans that had limitations on the number of family members allowed (non-slum β = 0.44, P < 0.05; slum β = 0.36, p < 0.05). Households in non-slum communities, in particular, had a high preference for health insurance plans that covered chronic illnesses and major surgeries to other plans (0.97 β, P < 0.05). Our findings suggest that location of the household influences willingness to pay with households from non-slum communities willing to pay more for the preferred attributes.

**Conclusion:**

Potential health insurance schemes should consider including both private and public health care providers and allow more household members to be enrolled in both slum and non-slum communities. However, the inclusion of more HH members should be weighed against the possible depletion of resources and other attributes. Potential health insurance schemes should also prioritize coverage for chronic illnesses and major surgeries in non-slum communities, in particular, to make the scheme attractive and acceptable for these communities.

**Supplementary Information:**

The online version contains supplementary material available at 10.1186/s12962-021-00274-8.

## Introduction

According to the sustainable development goal 3, target 8, member countries of the United Nations agreed to achieve universal health coverage (UHC) including: financial risk protection; access to quality essential health care services and; access to safe, effective, quality, and affordable essential medicines and vaccines for all by 2030 [1]. Low and middle-income countries face challenges in achieving UHC due to limited public resources, inefficient resource allocation, reliance on out-of-pocket (OOP) expenditures, and large population sizes [2]. For example, the current health expenditure as a percentage of gross domestic product (GDP) for sub-Saharan Africa is estimated at 5.2%, which is almost 50% lower than the global average (9.9%). In Uganda, the health expenditure as a share of the national GDP is almost 70% and 50% lower than the estimates for North America (16.6%) and the European Union (9.9%), respectively [3]. The Uganda government’s health expenditure as a share of its GDP and its health expenditure per capita are estimated at 0.97% and USD (United States dollars) 38.4, respectively. These estimates are less than the Working Group on Health Financing’s recommended health sectors’ share of GDP and health expenditure per capita that are expected to be at least 5% and USD 86, respectively [3–5].

The lower health sector’s share of the national GDP in Uganda indicates inadequacies in health financing, which may explain why 40% of the country’s health expenditure is OOP expenditure. The OOP expenditures in Uganda are two times higher than the World Health Organization (WHO)’s recommendation of 20% of the total health expenditure in a country [2, 4, 5]. This calls for more financial risk protection for the HHs in Uganda to protect them against OOP expenditures and their consequences (catastrophic health expenditure and impoverishment) [2]. Taxes and health insurance are the means recommended by the WHO to raise funds for the health sector in low and middle-income countries in order to achieve UHC (including financial risk protection) [2, 6]. Taxes and health insurance have shown good results in other African countries such as Ghana and Rwanda providing increased financial risk protection and increased service utilization [7, 8].

Uganda is in discussions to introduce a National Health Insurance Scheme (NHIS) in order to reduce OOP expenditures and raise adequate funds for its health sector [9]. The existing health insurance schemes in the country are mainly provided by the private sector and contribute less than 3% of the total health financing in the country and are often accessed by those in formal employment [5]. There has been some success with community health insurance schemes that usually target rural communities––indicating the communities’ interest in saving for health. However, these community health insurance schemes are usually based on subscription membership fees that are often low and cannot cover long hospitalizations and severe illnesses [10, 11].

A successful NHIS requires a scheme that has been well thought out and that reflects HHs’ preferences. In this study, we assessed HHs’ preferences and WTP for health insurance. Specifically, we focused on: (1) identifying differences between the HHs in slum and non-slum communities that may influence their choice of health insurance plan; (2) determining preferences for the attributes of health insurance by HHs and; (3) estimating WTP for health insurance by HHs. We applied a discrete choice experiment (DCE) approach that is different from the contingent valuation methods used in the prior studies conducted in Uganda. Contingent valuation methods can assess the total value of a commodity including the passive-use value but cannot determine the importance and the value HHs attach to the health insurance attributes [12–15]. Understanding the importance and value HHs attach to the different attributes of health insurance is critical in informing the design of an acceptable health insurance scheme [16–18].

## Methods

### Study design and study area

**Fig. 1:**
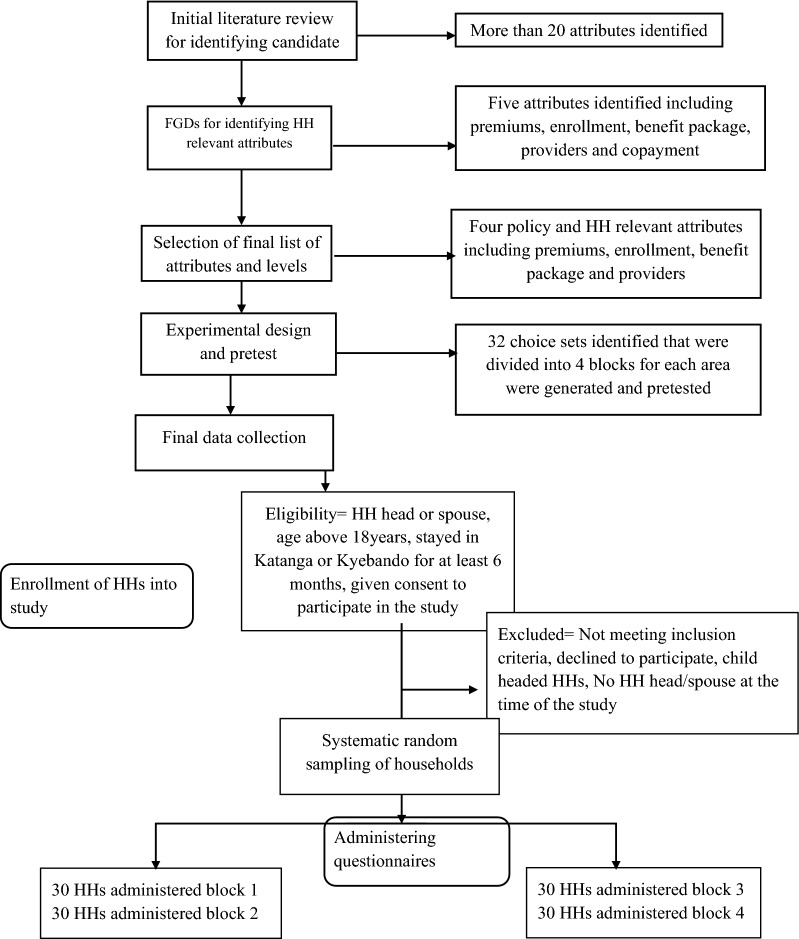
Study design

This was a DCE that was conducted from 16th February 2020 to 10th April 2020 in two parishes of the Kawempe division of Kampala capital city of Uganda. The Katanga slum represented the slum communities, while the Kyebando parish represented the non-slum communities in Kampala [19]. We chose this study setting because Kampala is the most populous urban center in the country with over 1.5 million people (31% of the urban population in the country); has more public and private health care facilities including several specialized public health care facilities compared with any other district in the country and; has a representation of the many tribes from all over the country [20–22]. We decided to stratify the study area into slum and non-slum communities in order to get a representative sample of the City, which has over 60% of the population living in slums [23]. Finally, we chose to conduct the study in the Kawempe division because of budget constraints and because we assumed that the population in the City is mainly divided into slum and non-slum dwellers. Figure 1 describes the study design.

### Development of the DCE

#### Choosing attributes and levels

In line with recommendations, a two-stage approach was used to generate attributes and levels [24–28]. First, conceptual attributes were identified from the literature and policy documents. We identified over 20 attributes from the literature review. The candidate attributes from the literature review that were relevant to Uganda included: premiums; the unit of enrollment; the type of health care providers; the service benefits package and; the copayment levels [9, 16, 29–35]. These candidate attributes were used to guide the design of the FGD guide (Additional file 1). Second, four focused group discussions (FGDs) were conducted in each area to ensure that the identified attributes from the literature were relevant to the HHs and to identify new ones. We probed participants on factors that would influence them to join a health insurance plan. We asked them specific questions about the potentially relevant attributes that we had earlier identified from the literature review. Participants were also asked to rank the attributes in order of importance. The FGDs were carried out by the first author with assistance from one research assistant in Luganda and English. We audio-recorded and later transcribed the data verbatim. We then translated the transcripts into English. We analyzed the data using both deductive and inductive thematic approaches. The main themes from the FGDs were related to premiums, providers of health services, unit of enrollment, and service benefits packages.

Premiums: The Ministry of Health (MoH) for Uganda proposed that self-employed individuals contribute UGX 100,000 (USD 26.7) (based on currency conversion rates from the OANDA website) [36] annually to the scheme, while salaried employees contribute 4% of their salary to the scheme [9]. The 4% is equivalent to UGX 68,000 (USD 18.1) to UGX 450,000 per year (USD 120) (based on average income ranges from the Uganda national household survey 2016/2017) [36, 37]. The employers would then be required to provide an additional 1% of the employee’s salary (in addition to the 4% contribution by the employee) [5, 37]. From the FGDs, participants from the slum area suggested premiums that ranged from UGX 20,000 (USD 5.3) per year to UGX 100,000 (USD 26.7) per year, while participants from the non-slum area suggested premiums that ranged from UGX 100,000 (USD 26.7) per year to UGX 300,000 (USD 80.0) per year. Therefore, for this attribute, we included levels that ranged from UGX 20,000 per year to UGX 100,000 per year for the slum community and UGX 100,000 per year to UGX 300,000 per year for the non-slum community.

Providers: From the literature, this attribute was mainly categorized depending on the way the health systems from the countries where the studies originated were organized. In some countries like Iran and Thailand, there were no faith-based health care providers while in Africa, they were common [29, 30, 32, 33, 38]. The majority of participants in the FGDs from both the slum and non-slum communities wanted to receive health services from both public and private health care providers, while a minority wanted to receive health services from private health care providers only. Therefore, for this attribute, we included the following levels: private health care providers only; both private and public health care providers and; public health care providers only. The private health care providers in our study included both private and faith-based health care providers.

Unit of enrollment: From the literature review, this attribute was categorized based on the type and number of family members the insurance plan is responsible for. For example, it was categorized by some researchers as self only, self plus spouse, self plus spouse plus children, and self plus extended family [29, 33, 34]. For the case of Uganda, the MoH proposed that the beneficiaries include the individual who pays for the health insurance plan, their spouse, and one of their children below the age of 18 years [9]. From the FGDs, the majority of participants were not interested in any health insurance plan that did not cover extended family members. The Majority of participants concerned with the depletion of resources suggested a restriction on the number of children enrolled to three per HH. Therefore, for this attribute, we considered the following levels: restricted enrollment (parents and their three children); unrestricted enrollment (parents without limitations on the number of children enrolled) and; extended family enrollment (parents, their children, and grandparents).

Service benefits package: From the literature review, this attribute was categorized as follows: basic, medium, and comprehensive benefits packages [29, 30] and; inpatient department services, outpatient department services, and drugs and tests [33]. From the FGDs, the majority of participants from the slum area pointed out that they were disproportionately affected by all diseases and therefore wanted all health conditions to be covered by the health insurance scheme. Others wanted some limitations on the type of health conditions covered by the health insurance scheme to avoid reckless use and depletion of resources. Participants from the non-slum community were equally divided with some wanting all health conditions covered, while others wanted some restrictions on the type of health conditions that health insurance covers. Therefore, in our study, this attribute included the following levels: simple service benefits package; moderate service benefits package and; comprehensive service benefits package. These attribute levels are defined in Table 1.

**Table 1:** Summary of attributes and levels

Attribute	Description	Regression label	Non-slum community	Slum community
			Levels	Levels
Premiums^a^	The yearly cost of the health insurance plan	Premiums	1. UGX 100,000 (USD 26.7) 2. UGX 200,000 (USD 53.3) 3. UGX 300,000 (USD 80.0)	1. UGX 20,000 (USD 5.3) 2. UGX 50,000 (USD 13.3) 3. UGX 100,000 (USD 26.7)
Providers^b^	The kind of health providers from which households will receive services	1. Private 2. Both 3. Reference level	1. Private providers only 2. Public and private providers 3. Public providers	1. Private providers only 2. Public and private providers 3. Public providers
Enrollment^c^	The type and number of family members that health insurance is responsible for	1. Restrict 2. Extend 3. Reference level	1. Restricted enrollment of children to 3 2. Extended family 3.Unrestricted enrollment of children	1. Restricted enrollment of children to 3 2. Extended family 3. Unrestricted enrollment of children
Health Service benefits package^ds^	The type of health services covered by the health insurance plan	1. Simple 2. Reference level 3. Comprehensive	1. Simple service benefits package 2. Moderate service benefits package 3. Comprehensive service benefits package	1. Simple service benefits package 2. Moderate service benefits package 3. Comprehensive service benefits package

^a^1 USD = 3750 UGX (April 2020 exchange rate)

^b^Public providers include health facilities that are owned by the government; private providers include health facilities that are owned by private individuals and faith-based organizations

^c^Restricted enrollment of children includes parents and only three children; unrestricted enrollment of children includes parents with no restrictions on the number of children and; the extended family includes children, their parents, and grandparents

^d^Simple service benefits package includes primary health care diseases, minor surgeries, ANC, family planning; moderate service benefits package includes chronic illnesses for example asthma, hypertension, and diabetes, major surgeries including caesarian sections and; comprehensive service benefits package includes cancers, advanced kidney diseases, heart surgeries, neurosurgeries, ICU, and special care units

#### Development of the DCE questionnaire

We used experimental design methods to derive choice sets. From the literature, two approaches are commonly used to design choice sets namely: orthogonal methods and D-optimal methods [39]. We used D-optimal methods because they perform better than orthogonal designs and require fewer choice sets and sample sizes compared to orthogonal designs [26]. The generated designs had 32 choice sets that were organized into 4 blocks to reduce the cognitive burden on each respondent. Each HH head/spouse answered 9 choice sets that included a fixed choice question that was not part of the experimental design and was used to assess the predictive power of the models.

Before the final data collection, we pilot tested the questionnaires on a total of 30 HHs (15 HHs from each area) and made minor revisions to the questionnaires. The DCE questionnaire collected data on the characteristics of the HHs, the choice sets, and the socio-economic status of the HHs.

**Fig. 2:**
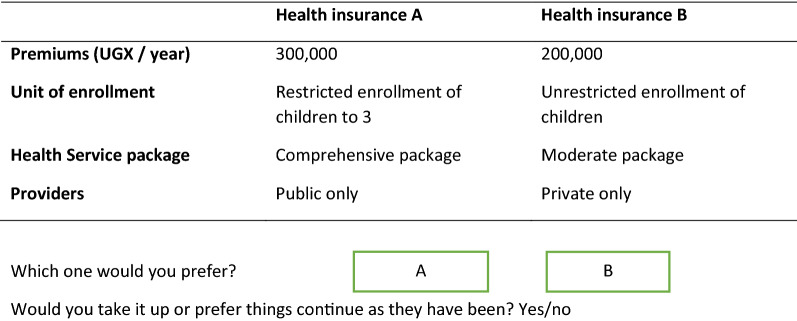
Example of a choice set

Figure 2 shows an example of a choice set. The data collection tools were in English and Luganda (the most commonly spoken languages in the area).

### Study population, sample size, study variables, sampling procedure, and data collection

We targeted HH heads/spouses in the Kawempe division of Kampala city who had stayed for at least 6 months in the area and were at least 18 years of age at the time of conducting this study. We left out child-headed HHs and HHs whose heads/spouses refused to participate or were not available at the time of conducting the study.

We used Louviere et al.’s [40] sample size calculator and determined that a minimum of 54 HHs were required for each area considering each HH head/spouse answered 8 choice sets; the choice probability in the population was 0.5; the allowed deviation from the true population proportion was 0.1 and; the confidence level was 95%. Considering that each version of the questionnaire is supposed to be answered by at least 30 HHs to avoid imprecise estimations during econometric analysis, the final sample size for each area was revised to 120 HHs such that each of the 4 versions of the questionnaire for each area was answered by at least 30 HHs [26]. The dependent variable was the choice of health insurance plan. The independent variables were the attributes. Table 2 shows all the variables that were measured and their description.

**Table 2:** Summary of variables

Variables	Description	Measurement
Premiums	The yearly cost for the health insurance plan in Ugandan shillings	Cost in Ugandan shillings
Enrollment^a^	The type of family members allowed by the health insurance plan	Restricted enrollment of children to 3 = 1 Unrestricted enrollment of children = 2 Extended family = 3
Service benefits package^b^	The type of benefit packages the health insurance plan offers	Simple benefits package = 1 Moderate benefits package = 2 Comprehensive benefits package = 3
Providers^c^	The type of providers for the health services	Public providers = 1 Private providers = 3 Both public and private = 2
Sex	The biological sex of the HH head	Male = 1, female = 0
Age	The age of the HH head in completed years	Age in completed years
Child categories	The number of children in the HH in the following categories	Below 5 Between 5 and 10 Between 10 and 19 Above 19
Pay health	History of paying OOP for health care in public facilities or paying for health products/services in private facilities because they were not available in public facilities	Yes = 1 No = 0
Marriage status	The marriage status of the HH head	Single = 1 Married = 2 Widowed = 3 Divorced = 4 Living together or cohabiting = 6
Occupation	The main occupation of the HH head	Farmer = 1 Salaried = 2 Casual worker = 3 Retired = 4 Trade/self-employed = 5 Unemployed = 6 Student = 7 Others = 8
Education level	The highest level of education for the HH head	No education = 1 Primary education = 2 Secondary education = 3 Post-secondary education = 4 Vocational education = 5
Possession of health insurance	Any possession of health insurance by the HH	Yes = 1 No = 0
Responsible organization	The arrangement for payment of health insurance by the HH	Private = 1 Employer based = 0
The usual source of health care	The usual providers of health care services for the HH members	Private = 1 Public = 2
Chronic illness in HH	Any member of the HH with chronic illness	Yes = 1 No = 0
Socio-economic status	The classification of HHs into socio-economic classes The assets were modified from the Uganda demographic and health survey questionnaire [35]	By principle component analysis and wealth index calculation using HH assets. We considered assets that included ownership of a sofa set, sideboard, wall watch, house ownership, type of fuel for cooking, floor material, wall material, source of drinking, type of toilet facility, sharing of toilet facility, number of rooms for sleeping, ownership of a bike, ownership of a motorcycle, ownership of a car, ownership of a bank account, number of transactions

^a^Restricted enrollment of children includes parents and only three children. Unrestricted enrollment of children includes parents with no restrictions on the number of children. The extended family includes children, their parents, and grandparents

^b^Simple service benefits package includes primary health care diseases, minor surgeries, ANC, and family planning; moderate service benefits package includes chronic illnesses for example asthma, hypertension, and diabetes, major surgeries including caesarian sections; comprehensive service benefits package includes cancers, advanced kidney disease, heart surgeries, neurosurgeries, ICU, and special care units

^c^Public providers include health facilities that are owned by the government; private providers include health facilities that are owned by private individuals and faith-based organization

We used face-to-face interviews to collect the data. We purposively selected the Kyebando parish to represent the non-slum communities and the Katanga slum to represent the slum communities. We selected the Kyebando parish to represent the non-slum communities because it has several gated residential houses with perimeter walls which are not typical of slum communities [19]. We then applied systematic sampling in the selection of the HHs that participated in the DCE from each area [41, 42]. We selected the reference HHs for each community randomly and thereafter interviewed every 29th HH in the slum community and every 20th HH in the non-slum community. The HH intervals for each community were obtained by dividing the estimates of the number of HHs in each area by the required sample sizes for each area. In case the approached HH did not meet the inclusion criteria, the next immediate HH would be approached and the process would be continued until the required sample size was obtained. The questionnaires from each block were distributed randomly to each of the interviewers daily. We edited the data in the field to ensure completeness and consistency.

### Data analysis

We chose the HH as the unit of analysis because in our study setting, especially among poorer HHs, decisions on seeking health care are usually based on HHs rather than individuals [30]. We used Chi-square tests and t-tests to assess for differences between the slum and the non-slum communities that may influence their choice behavior. Statistical analysis of the DCE data was based on random utility models**.** Random utility models are used to analyze the choices of individuals among discrete sets of alternatives.

Equation 1 shows the random utility model. According to the random utility theory, the utility that a HH (n) obtains from an insurance plan $i$ is given by:

1 $$U\_(in )=V(x\_in,B)+C\_in=V(x\_in,B)+\mu \_i+\varepsilon \_n$$

where $U\_in$ is the latent utility for an item $i$, V ($X\_in,B$) is the systematic component which is a function of attributes $x$ and marginal utility $B$. The total error term ($C\_in)$ in mixed logit models is split into two parts. The first part $\mu \_i$ is an individual-specific random effect that takes into account the panel nature of the data. The second part $\varepsilon \_n$ is an independent extreme value type-1 distributed part of the error such that the parameters can be estimated as a logit model [43, 44].

Equation 2 shows the form of the models.

2 $$V\left({x}_{in},B\right) \, = \, {B}_{0} + {B}_{1}premiums+{B}_{2}Both+{B}_{3}Private+{B}_{4}simple+{B}_{5}comprehensive+{B}_{6}Restrict+{B}_{7}Extended$$

where $B$ represents the parameter mean and its standard deviation in the population. All regression labels are defined in Table 1.

#### Calculating willingness to pay

WTP and its confidence interval were calculated using the “wtp” command in STATA 14.2 software using the delta method. WTP was calculated after estimating the model in Eq. 2 with the premium attribute fixed [45].

#### Validity and predictive power of the models

Theoretical validity refers to the extent to which the results are consistent with the expectations [46, 47]. Theoretical validity was assessed by checking the direction of association for some of the parameters. For example, the premium attribute was expected to have a negative coefficient (because people do not want to pay more for the same thing). Content validity for a DCE refers to the extent to which the attributes and levels are relevant to the participants [46, 48]. Content validity was ensured through careful development of the questionnaire that involved a thorough literature review to guide the development of the FGD guide and conducting FGDs with HH heads/spouses such that the generated attributes and levels are relevant to the communities. We also conducted pilot tests of the questionnaire.

To assess the predictive power of the models, we included one fixed choice set for each area (which was not part of the experimental design) that was answered by all participants. The preference estimates from the choice sets that were part of the experimental design were then used to estimate the probability of choosing one of the alternative health insurance plans in the fixed choice set.

### Ethical considerations

The institutional review board of the School of Public Health, Makerere University approved this study. Informed consent was secured from each person interviewed.

## Results

### Characteristics of households

Table 3 summarizes the characteristics of the HHs that participated in this study. All participants who agreed to participate in this study completed the questionnaire. Overall, 240 HH heads/spouses were interviewed––120 HH heads/spouses from the slum community and 120 HH heads/spouses from the non-slum community. The majority of HHs were headed by males (70.4%), 65% had at most three children, 88.7% reported paying for health services in public health care facilities, 94.0% were not insured, and 33.6% had at least one family member with a chronic illness. Also, 67.9% of HH heads were either married or living with a partner and 66.7% had attained at least a senior 4 level of education (equivalent to grade 11 in the United States of America education system) [49].

**Table 3:** Characteristics of households that participated in the discrete choice experiment

Variable	Slum community	Non-slum area community	Total
	Frequency (percentage)	Frequency (percentage)	Frequency (percentage)
Sex			
Female	34 (28.3)	37 (30.8)	71 (29.6)
Male	86 (71.7)	83 (69.2)	169 (70.4)
Age	Mean 32.49 (SD 8.2)	Mean 37.29 (SD = 10.6)***	Mean = 34.89 (SD = 9.8)
Marriage status			
Married	79 (65.8)	84 (70.0)	163 (67.9)
Not married	41 (34.2)	36 (30.0)	77 (32.1)
Number of HH with children			
0–3	88 (73.3)	68 (56.7)	156 (65.0)
> 3	32 (26.7)	52 (43.3)***	84 (35.0)
History of paying for health in government facilities			
Yes	107 (89.2)	106 (88.3)	212 (88.3)
No	13 (10.8)	14 (11.7)	28 (11.7)
Education level			
No education	5 (4.2)	5 (4.2)	10 (4.2)
Primary education	44 (36.7)	24 (20.0)	68 (28.3)
Senior 4	32 (26.7)	49 (40.8)	81 (33.8)
Senior 6	21 (17.5)	21 (17.5)	42 (17.5)
University	13 (10.8)	16 (13.3)	29 (12.1)
Vocational institute	5 (4.2)	5 (4.2)***	10 (4.2)
Occupation			
Salaried employee	38 (31.7)	39 (32.5)	77 (32.1)
Casual worker	17 (14.2)	14 (11.7)	31 (12.9)
Self-employed	43 (35.8)	55 (45.8)	98 (40.8)
Unemployed	18 (15.0)	7 (5.8)	25 (10.4)
Others	84(72.0)	85(70.8)***	169 (70.4)
HH income per month	Mean 367,625 (SD 269,622.7)	Mean 615,750 (SD 645,989)***	Mean 491,687.5(SD = 510,226.2)
HH income per month categories (UGX)			
0–200,000	42 (35.0)	14 (11.7)	22 (9.2)
200,001–400,000	44 (36.7)	41 (34.2)	85 (35.4)
400,001–600,000	19 (15.8)	32 (26.7)	51 (21.3)
> 600,001	15 (12.5)	33 (27.5)****	48 (20.0)
Possession of health insurance			
Yes	8 (6.7)	7 (5.9)	16 (6.7)
No	112 (93.3)	113 (94.1)	224 (93.3)
The usual source of health care			
Public facilities	58 (48.3)	46 (38.3)	104 (43.3)
Private facilities	62 (51.7)	74 (61.7)***	136 (56.7)
Chronic illness in HH			
Yes	45 (37.5)	36 (30.0)	81 (33.8)
No	75 (62.5)	84 (70.0)***	159 (66.2)
Socio-economic status			
Quantile 1	44 (37.7)	16 (13.3)	60 (25.0)
Quantile 2	36 (30.0)	24 (20.0)	60 (25.0)
Quantile 3	28 (23.3)	32 (26.7)	60 (25.0)
Quantile 4	12 (10.0)	48 (40.0)****	60 (25.0)

P values for categorical values were based on chi-squared tests while for continuous variables, it was based on t-tests

Private facilities include facilities owned by private individuals and organizations and faith-based organizations

*p < 0.05, **p < 0.01, ***p < 0.001, Where asterisks do not exist, there was no statistically significant difference in the variable identified

Compared with the slum community, the non-slum community: had more HHs with more than three children (43.3% non-slum versus 26.7% slum, p < 0.001); had more HH heads with at least a senior 4 level of education (40.8% non-slum versus 26.7% slum, p < 0.001) and; had more HH heads who were self-employed (45.8% non-slum versus 35.8% slum, p < 0.001). In terms of socio-economic position, the non-slum community had more HHs in the richest quartile (40.0% non-slum versus 10.0% slum, p < 0.001), and had HH heads who were on average earning UGX 248,125 (USD 66.2) (based on currency conversion rates from the OANDA website) [36] per month more than their slum counterparts (p < 0.001). In terms of presence in the HH of family members with chronic illnesses, the slum community had more HHs with members suffering from chronic illnesses compared with the non-slum community (37.5% slum versus 30.0% non-slum, p < 0.001).

### Preferences of health insurance attributes

Tables 4 summarizes HH preferences for the non-slum and slum communities. Two main effects models were fit to the data: one for the slum community; and the other for the non-slum community. Two models were fit to the data because of differences between the two communities in terms of characteristics that can influence choice behavior that included: HH characteristics, socioeconomic position, and presence in the HH of members with chronic illnesses. Additionally, the two sampled communities generated different levels for the premium attribute with the non-slum community suggesting higher premiums.

**Table 4:** Household preferences for health insurance attributes

Attribute	Non-slum community		Slum community	
	Mean (Standard error)	SD (Standard error)	Mean (Standard error)	SD (Standard error)
Premium (continuous*10,000 UGX /year)	− 0.21 (0.08)**		− 0.88 (0.18)***	
Enrollment^a^				
Extended family	0.44 (0.20)**	1.39 (0.26)***	0.36 (0.14)**	0.09 (0.60)
Restricted enrollment of children to 3 (Ref: unrestricted enrollment of children)	− 0.90 (0.16)***	0.70 (0.24)*	− 0.32 (0.17)*	1.00 (0.47)
Service benefits package^b^				
Simple	− 0.97 (0.22)***	1.62 (0.25)***	0.10 (0.29)	0.63 (0.30)
Comprehensive (Ref: moderate)	− 0.02 (0.14)	0.50 (0.29)	− 0.14 (0.18)	1.30 (0.23)***
Providers^c^				
Private only	− 0.08 (0.14)	0.46 (0.29)	− 0.06 (0.16)	0.92 (0.21)***
Private and public (Ref: Public)	0.81 (0.18)***	0.95 (0.24)**	0.87 (0.17)***	0.96 (0.22)***
Constant	0.02 (0.11)		0.09 (0.10)	
Model diagnostics				
Number of respondents	120		120	
Number of observations	1920		1920	
Loglikelihood	− 553.00		− 581.61	
Loglikelihood X^2	88.57		70.94	

The non-significant constant suggests that there is no left–right bias in the data. This means that respondents were not likely to choose the left than the right alternative

*p < 0.05, **p < 0.01, ***p < 0.001

^a^Restricted enrollment of children includes parents and only three children; unrestricted enrollment of children includes parents with no restrictions on the number of children and; the extended family includes children, their parents, and grandparents

^b^Simple service benefits package includes primary health care diseases, minor surgeries, ANC, and family planning; moderate service benefits package includes chronic illnesses (asthma, hypertension, and diabetes) and major surgeries including caesarian sections; comprehensive service benefits package includes cancers, advanced kidney disease, heart surgeries, neurosurgeries, ICU, and special care units

^c^Public providers include health facilities that are owned by the government; private providers include health facilities that are owned by private individuals and faith-based organizations

HHs in the non-slum community had a high preference for health insurance plans that allowed extended family enrollment (parents, their biological children, and grandparents) (β = 0.44, P < 0.05) as compared to plans that allowed an unrestricted enrollment of children (parents plus any number of their children) and; health insurance plans in which they could receive health care from both private and public health care providers as compared to plans in which they could receive health care from public health care providers only (β = 0.81, P < 0.05). HHs in the non-slum community had a low preference for health insurance plans that had a simple service benefits package (primary health care diseases, minor surgeries, ANC, and family planning) as compared to plans that had a moderate benefits package (chronic illnesses and major surgeries) (β = − 0.97, P < 0.05) and; health insurance plans that allowed a restricted enrollment of children (parents plus 3 children only) as compared to plans that allowed an unrestricted enrollment of children (β =  − 0.90, P < 0.05).

HHs in the slum community had a low preference for health insurance plans that allowed a restricted enrollment of children (parents plus 3 children only) (β = − 0.32, p < 0.05) but had a high preference for plans that allowed extended family enrollment (parents, grandparents, and biological children) as compared to plans that allowed an unrestricted enrollment of children (parents plus any number of their children) (β = 0.36, p < 0.05). HHs in the slum community also had a high preference for health insurance plans in which they could receive health care from both private and public health care providers as compared to plans in which they could receive health care from public health care providers only (β = 0.87, p < 0.05).

The significant standard deviations for the coefficients of the attributes show that the parameters vary in the population and there is preference heterogeneity among respondents for the health insurance attributes (a possible indication of unmeasured variables influencing preference estimates such as taste differences) [50, 51]. The premium attribute was modeled as a fixed parameter to more easily calculate WTP [28].

### Willingness to pay

Table 5 summarizes WTP estimates for the slum and the non-slum communities. HHs in the non-slum community were on average willing to pay: UGX 206,960 (USD 55.2) (based on currency conversion rates from the OANDA website) [36] per year for a plan that allowed extended family enrollment rather than one that allowed an unrestricted enrollment of children; UGX 418,932 (USD 111.7) per year for a plan that allowed an unrestricted enrollment of children rather than one that allowed a restricted enrollment of children; UGX 454,727 (USD 121.3) per year for a plan with a moderate service benefits package rather than one with a simple service benefits package and; UGX 377,057 (USD 100.5) per year to receive health care from both private and public health care providers rather than public health care providers only.

**Table 5:** Willingness to pay

Attributes	Non-slum community	Slum community
	WTP (95% CI)	WTP (95% CI)
Premium		
(continuous*UGX 100,000 per year)		
Enrollment^a^		
Restricted enrollment of children to 3		
Extended family	− 4.19 (− 7.33, − 1.05)	− 0.37 (− 0.76, 0.03)
(Ref: unrestricted enrollment of children)	2.07 (− 0.23, 4.37)	0.41 (0.05, 0.77)
Service benefits package^b^		
Simple		
Comprehensive	− 4.55 (− 8.26, 0.84)	0.12 (− 0.21, 0.45)
(Ref: moderate)	− 0.09 (− 1.39, 1.22)	− 0.15 (− 0.57, 0.26)
Providers^c^		
Private and public		
Private only	3.77 (0.79, 6.75)	0.99 (0.49, 1.48)
(Ref: Public)	0.38 (− 0.95, 1.71)	− 0.07 (− 0.42, 0.29)

^a^Restricted enrollment of children includes parents and only three children; unrestricted enrollment of children includes parents with no restrictions on the number of children and; the extended family includes children, their parents, and grandparents

^b^Simple service benefits package includes primary health care diseases, minor surgeries, ANC, and family planning; moderate service benefits package includes chronic illnesses (asthma, hypertension, and diabetes) and major surgeries including caesarian sections; comprehensive service benefits package includes cancers, advanced kidney disease, heart surgeries, neurosurgeries, ICU, and special care units

^c^Public providers include health facilities that are owned by the government; private providers include health facilities that are owned by private individuals and faith-based organizations

HHs in the slum community were on average willing to pay: UGX 36,540 (USD 9.7) per year for a plan that allowed an unrestricted enrollment of children rather than one that allowed a restricted enrollment of children; UGX 40,937 (USD 10.9) per year for a plan that allowed extended family enrollment rather one that allowed an unrestricted enrollment of children and; UGX 98,738 (USD 26.3) to receive health care from both private and public health care providers rather than public health care providers only.

### Assessing the validity of the results and predictive power of the models

Most of the estimated coefficients including the coefficient for the premium attribute had the expected signs. The model for the non-slum community predicted that 66.1% of the HH heads from the non-slum community would choose health insurance A of the fixed choice question. From the data collected, 64.2% of the HHs in the non-slum community chose health insurance A of the fixed choice question. The model for the slum community predicted that 54.5% of the HHs from the slum community would choose health insurance A of the fixed choice question. From the data collected, 54.2% of the HHs from the slum community chose health insurance A of the fixed choice question. These results show that both models had good prediction.

## Discussion

We present data of HH preferences and WTP for health insurance in the Kawempe division of Kampala city stratified into slum and non-slum communities. To our knowledge, this is the first study from Uganda that has examined preferences for health insurance attributes and their valuation by HHs. We found differences in HH characteristics between slum and non-slum communities, which may influence their health access choices that ultimately affect their choice of health insurance plans. Although we found differences between the two communities in terms of preferences for the service benefits package, some similarities were identified. For instance, we found that HHs in both communities preferred health insurance plans that have fewer restrictions on the number of family members allowed and health insurance plans in which they can receive health care from both private and public health care providers.

HHs in both communities preferred health insurance plans that allow extended family enrollment. In Africa, most families are extended families. Therefore, plans that allow extended family enrollment are expected to be preferred as has been indicated in other studies [16, 33, 34]. However, one study that was conducted in Ethiopia had different results with HHs preferring limited family enrollment [33]. The authors found these results surprising but contend that the respondents may have attached costs to enrolling extended family members, or the cost of health care for their parents may have been shared among siblings. Thus, some of the interviewed participants may not have been directly responsible for the health of their parents [33]. Contrary to our expectations, HHs in the slum community had a similar preference for health insurance plans that allow a restricted enrollment of children and plans that allow an unrestricted enrollment of children: this may have been because HHs in the slum community had fewer children compared with those in the non-slum community. The greater number of children in the HHs from the non-slum community compared with the slum community was also not expected. A possible explanation for this finding could be due to the HH heads in the slum community being younger and as their families grow, and possibly their finances improve, they move out of the slum communities. Including more HH members in the NHIS would be attractive for the majority of HHs in both communities but this needs to be weighed against the other attributes and the possible depletion of resources because of increased demand.

Regarding the service benefits package, HHs in the non-slum community had a high preference for health insurance plans that had a moderate service benefits package (chronic illnesses and major surgeries) as compared to plans that had either a simple service benefits package (primary health care diseases, minor surgeries, ANC, and family planning) or; a comprehensive service benefits package (cancers, kidney disease, heart surgeries, neurosurgeries, ICU, and special care units). As indicated in other studies, the preference for the service benefits packages depends on the community’s perceptions about the costs and the seriousness of the health conditions covered by health insurance [30, 52], For instance, in our study, the health conditions under the moderate benefits package are often perceived to be more serious and more costly compared with health conditions under the simple benefits package. For the case of the comprehensive benefits package, the health conditions covered may have been perceived by the HHs to be rare and therefore not a priority to insure against (possibly because few family members are likely to require management for them). Our results agree with findings from other DCEs in which coverage for major surgeries and chronic illnesses was important for the respondents [16, 30]. In contrast, HH from the slum community equally preferred all forms of the service benefits packages. This may be explained by the fact that slum communities are disproportionately affected by most diseases compared with the rest of the communities and therefore, it was hard for them to decide on the best benefits package. Therefore, the MoH may need to modify the scheme for poor communities.

Furthermore, HHs in both communities preferred plans in which they can receive health care from both private and public health care providers. These findings agree with several other studies that found that, given the opportunity, individuals prefer to receive health care from both private and public healthcare providers [33, 38]. The preference for both providers in our study is complex because of the various health system issues. For instance, on one hand, the public is aware of the public health facilities’ challenges such as health worker absenteeism, low staffing, frequent breakdown of equipment due to poor maintenance, and unavailability of drugs. On the other hand, the public health care facilities, particularly the referral hospitals, are known to have better infrastructure and more qualified staff [53, 54]. The preference for both providers may also be explained by the HH’s concerns about the profit-making motives of private health care providers that may compromise their services [35, 54]. Therefore, the MoH may need to contract with some private providers in addition to public providers in NHIS to make the scheme more attractive and acceptable for the majority of HHs.

Our study shows that HHs in the non-slum community are willing to pay more for the preferred attributes compared with their counterparts in the slum community. This is not surprising and can be explained by a higher income and awareness level of the benefits of health insurance in the non-slum community compared with the slum community [30, 52]. For instance, in our study, the non-slum community had more HH heads who had attained at least a Senior 4 level of education (equivalent to grade 11 in the United States of America education system) [49] compared with the slum community. The higher education attainment by HH heads in the non-slum community may have helped them to earn more income and have a higher level of awareness for the benefits of health insurance [30]. As indicated in other studies, the higher the level of education, the greater the WTP for health insurance [13, 52].

This study also assessed the validity of the estimated models and found that most of the coefficients including the premium attribute had the expected signs. The premium attribute had a negative sign in both models which shows that as the premiums increased, the preferences for health insurance plans decreased [16]. This adds to the theoretical validity of the results.

## Strengths and limitations of this study

The strength of this study––based on the design––is that DCEs not only estimate HH preferences for the attributes of health insurance, but can also be used to estimate valuation of the attributes, and thus, are not as limited as contingent methods that can estimate WTP for health insurance but not attributes’ preferences.

The limitation of this study––based on the study design––is that the respondents may face challenges in answering the multiple questions that require tradeoffs characteristic of DCEs.

In terms of generalizability, our findings may apply to Kampala city because the residents of Kampala are mainly divided into the slum and non-slum dwellers. However, these results may also apply to other urban populations of Uganda with similar contexts as Kampala. The results from the slum community may also reflect rural preferences and willingness to pay since most of the slum dwellers are usually immigrants from rural communities [55, 56].

## Conclusions and recommendations

Our study reveals that HHs in the slum and the non-slum communities prefer and value plans that include both private and public health care providers. Therefore, for the type of health care providers to be included in the NHIS, we recommend that the MoH considers contracting with some private health care providers to make the scheme more attractive and acceptable for the majority of HHs. Although findings from our study suggest that more HHs may join the NHIS if more family members are allowed, this needs to be weighed against possible depletion of resources. For the service benefits package to include in the NHIS, we advise the MoH to prioritize providing coverage for chronic illnesses and major surgeries especially for the non-slum communities rather than prioritizing specialized care units. We recommend that the MoH subsidizes the scheme for the poor communities (slum and rural) to make the scheme affordable for the poor while at the same time set reasonable premiums for the non-slum communities to raise adequate funds for the health sector.

Further research may be needed targeting specifically people who work in the formal sector and the richest class to provide information about these groups’ preferences and WTP. Qualitative research targeting policymakers in the MoH, the health workers, the district health teams and, the legislators may also be needed to get their perspectives on how the health insurance scheme should be designed.

## Supplementary Information

**Additional file 1.** FGD guide.

## Data Availability

Data is available from the corresponding author at reasonable request.

## Abbreviations

CI: Confidence interval
DCE: Discrete choice experiment
FGD: Focused group discussion
GDP: Gross domestic product
HH: Household
ICU: Intensive care unit
IPD: Inpatient department
MoH: Ministry of Health
MoPS: Ministry of Public service
NHIS: National health insurance scheme
OECD: Organization for Economic Cooperation and Development
OOP: Out of pocket
OPD: Outpatient department
SD: Standard deviation
UBOS: Uganda Bureau of Statistics
UGX: Uganda shillings
UHC: Universal health coverage
USD: United States dollars
WB: World Bank
WHO: World Health Organization
WTP: Willingness to pay
