# Supplementary material for: Household preferences and willingness to pay for health insurance in Kampala City: a discrete choice experiment
Source: Cost Eff Resour Alloc. 2021 Apr 20;19:21. doi: 10.1186/s12962-021-00274-8 (PMC8056698; doi:10.1186/s12962-021-00274-8)
Supplement: Supplementary file 1 — Additional file 1. FGD guide. [file 12962_2021_274_MOESM1_ESM.docx]

**Focused group discussions guide**

**Exploring attributes and attribute levels of health insurance**

**Basic information about FGD**

| FGD number |  |
| --- | --- |
| Starting time |  |
| Name of village |  |
| Sex |  |
| Closest health facility type |  |
| Number of participants |  |
| Name of facilitator |  |
| Closing time |  |

**Section 1: Introduction**

I am ……………………………………. from Makerere university. My colleague is…………………………………………., my research assistant. The discussion we wish to have with you today is about the national health insurance scheme that the government is designing. We would like to find out your opinions about it and how it should be designed to reflect the way you wish it to be designed.

Specifically, we shall be discussing the following topics:

- Your awareness, experience and understanding of health insurance.
- We shall then explain to what health insurance is all about.
- We shall then discuss with you the important characteristics that will influence your willingness to join this national health insurance scheme.
- We will then discuss the contributions you will be expected to make, the benefits you expect to get, the service providers you wish to be included and the degree of enrollment of household members.
- We will also identify and discuss the kind of health services you would like to be included in the benefit package.

As people who will be making contributions, it is important that your views are considered in the design of the national health insurance scheme. This is to ensure that it is more acceptable to you. I have not been sent by the ministry of health. I am doing this as part of my master’s program. However, I intend to inform the ministry of health about the findings. This discussion will last approximately 90 minutes.

**Section 2: Awareness, experience and understanding of health insurance.**

1. Have you ever heard of health insurance?
2. What do you think is the function of health insurance?
3. Are you members of any health insurance scheme?

*Probes-names of health insurance schemes, types of health insurance schemes e.g. employer based, community based.*

1. Can you tell us about what you know about these health insurance schemes?

*Probes-What kind of people are members?*

*Probe-Premium levels and how they are paid.*

*Probe- Health services covered.*

*Probe- Health providers who render services to clients*

1. For those who do not have health insurance, how do you pay for health care when you fall sick?

*Probes- OOP, free public services.*

*Probe-What kind of facility do you go to? Private or public, traditional healers*

1. What kind of health services do you normally pay for OOP and at what type of health facility?

*Probes -why do you pay for them*?

1. Are there services that you sometimes need but are not available or inaccessible at the health facilities? Why?

*Probes -Names of health services and why they are not available or not accessible.*

*Probes-Where they think they can obtain them.*

**Section 3: Introduction to health insurance**

Insurance is like buying an umbrella when it is not raining preparing for the period when it is likely to rain. This is because, when the rain comes, you may not have money to buy the umbrella or it may be too expensive to buy. The concept of health insurance is like this, people who are not sick (both poor and rich) put money in a common bucket and when any member falls sick, the collected funds can cater for the health care costs of the individual. The costs sometimes can be very high when compared to the money you contributed but health insurance will cover for it. If you contribute and you do not fall sick, no refund is given. So, it is like the entire community shares the risk of ill health.

The government is in the process of designing a national health insurance scheme for its population. This is because ever since the abolition of user fees in the early 2000s, it has been struggling to finance health care and this has led to poor quality services in the public health facilities.

1. Do you now understand what health insurance is?
2. Would you consider it useful in your community?
3. Would you be willing to contribute to such an arrangement if it is set up?

**Section 4: Derivation of attributes**

Based on what we have just discussed

1. Assume you had a choice to join or not to join, which characteristics of health insurance would you consider very important in determining your decision? Why?

*Probes -cost, benefit package, enrollment, service provision, copayment, etc.*

**Section 5: Deriving levels**

**Benefit package**

1. What kind of health services would you like to be covered under the health insurance scheme?

*Probe- why would you like them to be covered?*

1. Due to limitation of the amount of funds collected and possible irrational use of services, insurance schemes sometimes ask members to pay some amount or percentage before they can step in. What percentage would you be willing to pay?

*Probes -* *What percentage of the bill would you be willing to contribute? 90%, 50%, 25%*

1. Can you please order the services you would wish to be included by the order of importance as a group?

*Probe- Why for each service?*

**Service provision**

1. When you fall sick, where do you often go for health care? Why?

*Probes-private, public, traditional healers*

1. What kind of providers would you prefer to go to if you were covered by health insurance?

*Probes-private, public, both private and public, traditional healers.*

1. How would you like the health care providers to be assigned to you?

*Probes-private only, public only, both private and public, why?*

**Premiums/ enrollment**

1. How would you like the money for the health insurance scheme to be set?

*Probes -per individual, per household*

*Probes -flat rate or according to people’s earnings?*

1. If per HH, what kind of members should be included?

*Probes – self + spouse, self + spouse+ children up to age 18 years, self + spouse+ children+ extended family members in the HH.*

1. How often would you like the premiums to be collected from you? Why?

*Probes-weekly, monthly, annually*

*Probes -at what time?*

1. Present list of services. How much do you think would be a reasonable amount for the services you wish to be included in the benefit package? Why?

*Probes – individual vs HH.*

1. Who should be responsible for paying? Why?

*Probes- employer, individual, HH head*

1. How should the money be collected from you?

*Probes-cash vs in kind, Possibility of paying in installments*.

***Thank you very much for your contributions and time***
